# Radar-to-Lidar: Heterogeneous Place Recognition *via* Joint Learning

**DOI:** 10.3389/frobt.2021.661199

**Published:** 2021-05-17

**Authors:** Huan Yin, Xuecheng Xu, Yue Wang, Rong Xiong

**Affiliations:** Institute of Cyber-Systems and Control, College of Control Science and Engineering, Zhejiang University, Hangzhou, China

**Keywords:** radar, lidar, heterogeneous measurements, place recognition, deep neural network, mobile robot

## Abstract

Place recognition is critical for both offline mapping and online localization. However, current single-sensor based place recognition still remains challenging in adverse conditions. In this paper, a heterogeneous measurement based framework is proposed for long-term place recognition, which retrieves the query radar scans from the existing lidar (Light Detection and Ranging) maps. To achieve this, a deep neural network is built with joint training in the learning stage, and then in the testing stage, shared embeddings of radar and lidar are extracted for heterogeneous place recognition. To validate the effectiveness of the proposed method, we conducted tests and generalization experiments on the multi-session public datasets and compared them to other competitive methods. The experimental results indicate that our model is able to perform multiple place recognitions: lidar-to-lidar (L2L), radar-to-radar (R2R), and radar-to-lidar (R2L), while the learned model is trained only once. We also release the source code publicly: https://github.com/ZJUYH/radar-to-lidar-place-recognition.

## 1. Introduction

Place recognition is a basic technique for both field robots in the wild and automated vehicles on the road, which helps the agent to recognize revisited places when traveling. In the mapping session or Simultaneous Localization and Mapping (SLAM), place recognition is equal to loop closure detection, which is indispensable for global consistent map construction. In the localization session, place recognition is able to localize the robot *via* data retrieval, thus achieving global localization from scratch in GPS-denied environments.

**Figure 1:**
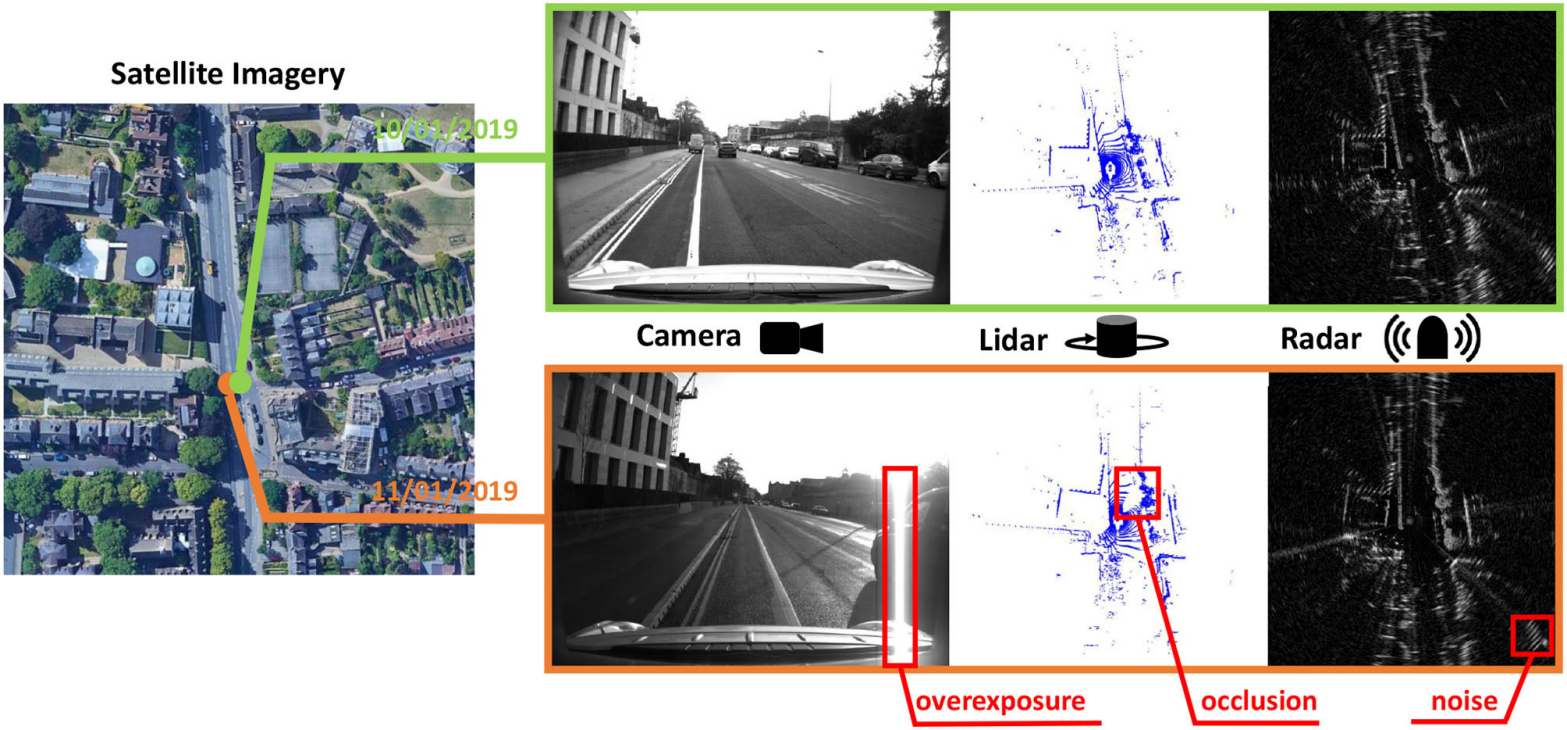
The sensor data collected at the same place but different time. These data are selected from the Oxford Radar RobotCar dataset. Obviously, every sensor has its weakness for long-term robotic perception.

Essentially, the major challenge for place recognition is how to return the correct place retrieval under the environmental variations. For visual place recognition (Lowry et al., 2015), the illumination change is the considerable variation across day and night, which makes the image retrieval extremely challenging for the mobile robots. As for lidar (Light Detection and Ranging)-based perception (Elhousni and Huang, 2020), the lidar scanner does not suffer from the illumination variations and provides precise measurements of the surrounding environments. But in adverse conditions, fog and strong light etc., or in highly dynamic environments, the lidar data are affected badly by low reflections (Carballo et al., 2020) or occlusions (Kim et al., 2020). Compared to the vision or lidar, radar sensor is naturally lighting and weather invariant and has been widely applied in the Advanced Driver Assistance Systems (ADAS) for object detection. But on the other hand, radar sensor generates noisy measurements, thus resulting in challenges for radar-based place recognition. So overall, there still remain different problems in the conventional single-sensor based place recognition, and we present a case study in Figure 1 for understanding.

Intuitively, these problems arise from the sensor itself at the front-end and not the recognition algorithm at the back-end. To overcome these difficulties, we consider a combination of multiple sensors desired for long-term place recognition, for example, building map database in stable environments, while performing query-based place recognition in adverse conditions. One step further, given that large-scale high-definition lidar maps have been deployed for commercial use (Li et al., 2017), a radar-to-lidar (R2L) based place recognition is a feasible solution, which is robust to the weather changes and does not require extra radar mapping session, thus making the place recognition module more applicable in the real world.

In this paper, we propose a heterogeneous place recognition framework using joint learning strategy. Specifically, we first build a shared network to extract feature embeddings of radar and lidar, and then rotation-invariant signatures are generated *via* Fourier transform. The whole network is trained jointly with the heterogeneous measurement inputs. In the experimental section, the trained model achieves not only homogeneous place recognition for radar or lidar, but also the heterogeneous task for R2L. In summary, the contributions of this paper are listed as follows:

- A deep neural network is proposed to extract the feature embeddings of radar and lidar, which is trained with a joint triplet loss. The learned model is trained once and achieves multiple place recognition tasks.
- We conduct the multi-session experiments in two public datasets, also with the comparisons to other methods, thus demonstrating the effectiveness of the proposed method in the real-world application.

The rest of this paper is organized as follows: section 2 reviews the related works. Our proposed method is introduced in section 3. The experiments using two public datasets are described in section 4. Finally, we conclude a brief overview of our method and a future outlook in section 5.

## 2. Related Works

### 2.1. Visual-Based Place Recognition

Visual place recognition aims at retrieving similar images from the database according to the current image and robot pose. Various image features have been proposed to measure the image similarities, such as SURF (Bay et al., 2006) and ORB (Rublee et al., 2011) etc. These features can also measure the similarities between pre-defined image patches (Filliat, 2007; Jégou et al., 2010). Based on these front-end descriptors, some researchers proposed probabilistic approach to Cummins and Newman (2008) or searched the best candidates in the past sequences (Milford and Wyeth, 2012). But due to the limitation of handcrafted descriptors, these visual place recognition methods are sensitive to the environmental changings.

With the increasing development of deep learning technique, more researchers build Convolutional Neural Networks (CNN) to solve the visual place recognition problem. Compared to the conventional descriptors, the CNN-based methods are more flexible on trainable parameters (Arandjelovic et al., 2016) and also more robust across the season changes (Latif et al., 2018). Currently, there are some open issues to be studied in the vision and robotics community, such as feature selection and fusion for visual place recognition (Zhang et al., 2020).

### 2.2. Lidar-Based Place Recognition

According to the generation process of representations, the lidar-based place recognition methods can also be classified into two categories, the handcrafted-based and the learning-based. Bosse and Zlot (2013) proposed to extract the three-dimensional (3D) keypoints from 3D lidar points and performed the place recognition *via* keypoint voting. In Dubé et al. (2020), local segments were learned from CNN to represent places effectively. Despite the local representations, several global handcrafted descriptors (He et al., 2016; Kim and Kim, 2018; Wang et al., 2019) or matching-based methods (Le Gentil et al., 2020) were proposed to solve the point-cloud-based place recognition. These global descriptors are based on the structure of the range distribution and efficient for place recognition. Similarly, some learning-based methods first generated the representations according to the statistical properties, then fed them into the classifiers (Granström et al., 2011) or CNN (Yin et al., 2019; Chen et al., 2020). In addition, some researchers proposed to learn the point features in an end-to-end manner recently (Uy and Lee, 2018; Liu et al., 2019), while these methods bring more complexity for network training and recognition inference.

### 2.3. Radar-Based Mapping and Localization

Compared to the cameras and laser scanners, radar sensor has already been used in the automotive industry (Krstanovic et al., 2012). With the development of Frequency-Modulated Continuous-Wave (FMCW) radar sensor^1^, the mapping and localization topics are studied in the recent years, for example the RadarSLAM (Hong et al., 2020), radar odometry (Cen and Newman, 2018; Barnes et al., 2020b), and radar localization on lidar maps (Yin et al., 2020, 2021).

For radar-based place recognition, Kim et al. (2020) extended the lidar-based handcrafted representation (Kim and Kim, 2018) to radar data directly. In Săftescu et al. (2020), NetVLAD (Arandjelovic et al., 2016) was used to achieve radar-to-radar (R2R) place recognition. Then, the researchers used sequential radar scans to improve the localization performance (Gadd et al., 2020). In this paper, a deep neural network is also proposed to extract feature embeddings, but the proposed framework aims at heterogeneous place recognition.

### 2.4. Multi-Modal Measurements for Robotic Perception

Many mobile robots and vehicles are equipped with multiple sensors and various perception tasks can be achieved *via* heterogeneous sensor measurements, for example, visual localization on point cloud maps (Ding et al., 2019; Feng et al., 2019) and radar data matching on satellite images (Tang et al., 2020). While for place recognition, there are few methods performed on heterogeneous measurements. Cattaneo et al. (2020) built shared embedding space for visual and lidar, thus achieving global visual localization on lidar maps *via* place recognition. Some researchers proposed to conduct the fusion of image and lidar points for place recognition (Xie et al., 2020). Similarly, in Pan et al. (2020), the authors first built local dense lidar maps from raw lidar scans, and then proposed a compound network to align the feature embeddings of image and lidar maps. The proposed framework was able to achieve bi-modal place recognition using one global descriptor. In summary, we consider the matching or fusion of multi-modal measurements as a growing trend in the robotics community.

## 3. Methods

**Figure 2:**
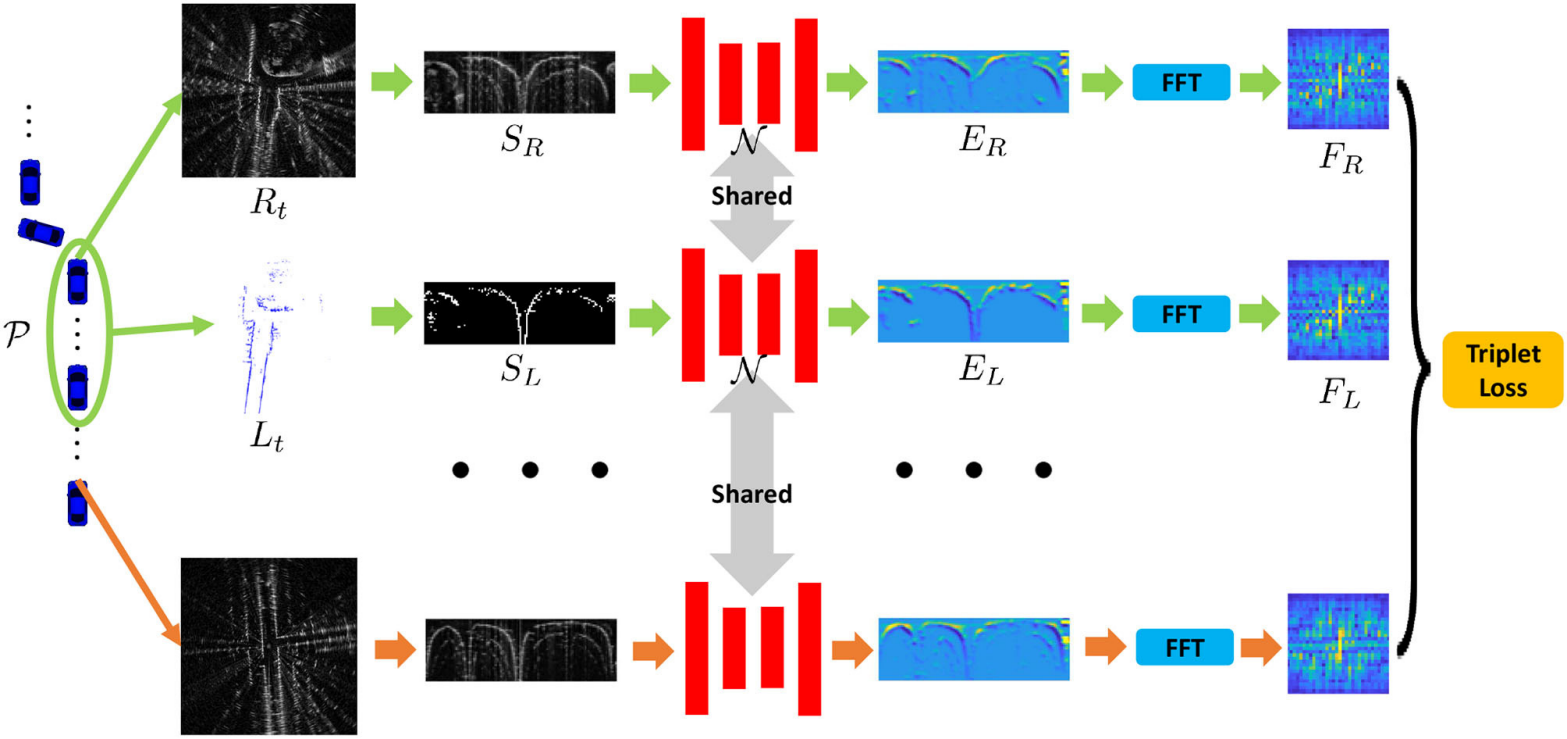
Our proposed framework to train the joint place recognition for radar and lidar data. The first and second rows indicate that the radar scan and the lidar submap are collected from the same place, while for the last row, it is regarded as a negative sample in the learning stage.

Our proposed framework is presented in Figure 2. There are several pipelines, including building lidar submaps, generation of the learned signatures, etc. Finally, the learned model generates low-dimensional representations for place recognition task in this paper.

### 3.1. Building Lidar Submaps

Generally, the detection range of radar is much longer than lidar. To reduce the data difference for joint learning, we set the maximum range as *r*_*max*_ meters in radar and lidar data. The 3D lidar also contains more height information compared to the 2D radar, and therefore we remove the redundant laser points and only keep the points near the radar sensor on Z-axis.

Despite the above difference, the lidar points are more sparse and easily occluded by other onboard sensors, and also by the dynamics on road. In this paper, we follow the experimental settings in Pan et al. (2020) and propose to build submaps by accumulating the sequential lidar data. Then, the problem turns to how many poses or lidar scans should be used for submap building. Suppose the robot travels at a pose *p*_*t*_, and we use the poses from the start pose *p*_*t*−*t*_1__ to the end pose *p*_*t*+*t*_2__, where *t*, *t*_1_, and *t*_2_ are time indexes. In order to keep the consistency of lidar submap and radar scan, we propose to achieve the *t*_1_ and *t*_2_ using the following criteria:

- The maximum euclidean distance from *p*_*t*−*t*_1__ to *p*_*t*_ is limited to *r*_*max*_ meters, guaranteeing the traveled length of the submap. It is same for the maximum distance from *p*_*t*_ to *p*_*t*+*t*_2__.
- The maximum rotational angle from *p*_*t*−*t*_1__ to *p*_*t*_ should not be greater than θ_*max*_, which makes the lidar submaps more feasible at the turning corners. The rotational angles for mobile robots and vehicles are usually the yaw angles on the ground plane. Also, it is same for the maximum yaw angle from *p*_*t*_ to *p*_*t*+*t*_2__.

On the other hand, the lidar submap is desired to be as long as radar scan. Based on the criteria above, we can fomulate the retrieval of *t*_1_ as follows:

(1) $$\begin{array}{l} \begin{array}{ll} \underset{t_{1}}{\text{minimize}} & t-t_{1}\\ \text{subject to} & {\left\|p_{t}-p_{t-t_{1}}\right\|}_{2}\leq r_{max}\\ \; & |∠p_{t}-∠p_{t-t_{1}}|\leq \theta_{max}. \end{array} \end{array}$$

where ∠ is the yaw angle of pose. It is maximize operation for *t*+*t*_2_ to achieve the *p*_*t*+*t*_2__ with similar constraints. Specifically, we use a greedy strategy to search *t*_1_ and *t*_2_, and we use the retrieval of *t*_1_ as an example in Algorithm 1. With obtained boundaries *t* − *t*_1_ and *t* + *t*_2_, a lidar submap can be built directly by accumulating the lidar scans from *p*_*t*−*t*_1__ to *p*_*t*+*t*_2__, thus achieving a lidar submap at the robot pose *p*_*t*_. Note that we use ground truth poses for the map building in this subsection.

**Algorithm 1:** Greedy search for *t*_1_ retrieval

**Require:**
The robot pose: *t*; The maximum euclidean distance: *r*_*max*_; The maximum rotational angle: θ_*max*_;
**Ensure:**
*t*_1_ = 1
**while** ||*p*_*t*_ − *p*_*t*−*t*_1__||_2_ ≤ *r*_*max*_ and |∠*p*_*t*_ − ∠*p*_*t*−*t*_1__| ≤ θ_*max*_ **do**
*t*_1_ = *t*_1_+1
**end while**

One might suggest that radar mapping should also be considered. However, as shown in Figure 1, there exist false positives and other noises in radar scans, resulting in inapplicable representations after radar mapping. In this context, we propose to build lidar submaps rather than radar submaps for heterogeneous place recognition in this paper.

### 3.2. Signature Generation

With the built lidar submaps *L*_*t*_ and the radar scan *R*_*t*_ collected at the same pose, we first use the ScanContext (Kim and Kim, 2018; Kim et al., 2020) to extract the representations *S*_*L*_ and *S*_*R*_, respectively. Specifically, for radar scan, the ScanContext is the polar representation essentially. As for the lidar point clouds, we follow the settings in our previous research (Xu et al., 2021), in which occupied representation achieves the best performance. Since the radar data are generated on 2D *x* − *y* plane, we use the single-layer binary grids as the occupied representation, which indicates that the height information is removed in this paper.

Then, we build network N to extract the feature embeddings *E*_*L*_ and *E*_*R*_. Specifically, *S*_*L*_ and *S*_*R*_ are fed into a shared U-Net architecture (Ronneberger et al., 2015), and our hidden representations *E*_*L*_ and *E*_*R*_ are obtained in the feature space *via* feed-forward network. One might suggest that the networks should be different for the heterogeneous measurements, but considering that there are commons between radar and lidar, we propose to use the Siamese structure to extract the embeddings. We validate this structure in following experimental section.

Finally, we follow the process in our previous work (Xu et al., 2021) and apply Fast Fourier Transformation (FFT) to the polar bird's eye view (BEV) representation. To make the process more efficient, we only extract the informative low-frequency component using the low-pass filter, and then signatures *F*_*L*_ and *F*_*R*_ are generated in the frequency domain. Theoretically, the rotation of vehicle equals to the translation of *E*_*L*_ and *E*_*R*_ in the polar domain, and the magnitude of frequency spectrum is actually, translation-invariant thus making the final signatures rotation-invariant to the vehicle heading. Overall, we summarize the processes of the signature generation as follows:

(2) $$\begin{array}{l} L_{t},R_{t}\begin{array}{c} \text{ScanContext}\\ \to \end{array}S_{L},S_{R}\begin{array}{c} N\\ \to \end{array}E_{L},E_{R}\begin{array}{c} \text{FFT}\\ \to \end{array}F_{L},F_{R} \end{array}$$

and the visualization of Equation (2) is presented in Figure 2. Essentially, the final signatures *F* are the learned fingerprints of places. If two signatures are similar, the two places are close to each other and vice versa.

### 3.3. Joint Training

With the generated batch $F=\left\{F_{L},F_{R}\right\}$, we propose to achieve the heterogeneous place recognition using the joint training strategy. Specifically, R2R, lidar-to-lidar (L2L), and R2L are trained together under the supervision of one loss function. To achieve this, we build the triplet loss and mix all the combinations in it, which is formulated as follows:

(3) $$\begin{array}{l} L_{1}=\frac{1}{\left|F\right|}\underset{F\in F}{\sum }\text{max}\left(0,m+\text{pos}\left(F\right)-\text{neg}\left(F\right)\right) \end{array}$$

where *m* is a margin value. *F* is any combination in the set F, for example, the combination of {anchor, positive, negative} samples can be {radar, lidar, radar}, or {radar, radar, lidar}. The number of these combinations for training is $|F|=2^{3}$. pos(*F*) is the measured Euclidean distance for a pair of positive samples, while neg(*F*) for the anchor and negative sample.

In this way, the trained model achieves not only the single-sensor based place recognition but also the homogeneous task for R2L. Note that there are three place recognition tasks in this paper, R2R, L2L, and R2L (or L2R), but we only train the network once.

## 4. Experiments

The experiments are introduced in this section. We first present the setup and configuration, then followed the loop closure detection and place recognition results, with the comparison to other methods. Case study examples are also included for better understanding of the result. Considering that most of the existing maps are built by lidar in the robotics community, the R2L task is more interesting and meaningful for heterogeneous place recognition, compared to lidar-to-radar task. Therefore, we only perform R2L to demonstrate the effectiveness of our proposed framework.

### 4.1. Implementation and Experimental Setup

The proposed network is implemented using PyTorch (Paszke et al., 2019). We set the maximum range distance as *r*_*max*_ = 80 m, and set $\theta_{max}=90^{{}^{\circ}}$. For the ScanContext representation, we set the size as 40 × 120 and finally, achieve the 32 × 32 low-frequency signature. Some parameters have an influence on the model performance, for example, bin sizes, and we follow the experimental settings in Xu et al. (2021), which have been demonstrated to be effective and efficient. In the training session, there are more than 8,000 samples generated randomly and the batchsize is set as 16. We run six epochs with the Adam optimizer (Kingma and Ba, 2015) and a decayed learning rate from 0.001.

**Figure 3:**
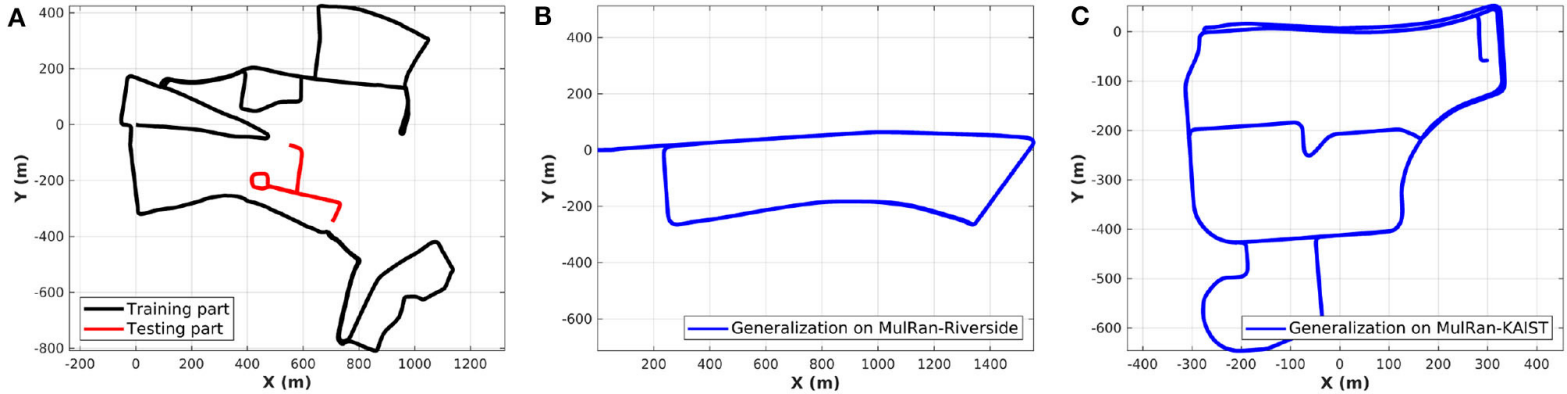
**(A)** We split the trajectory of RobotCar dataset into training and testing session. **(B,C)** The trained model is generalized to the Riverside and KAIST of the MulRan dataset for evaluation directly, which contain a driving distance near 7 and 6 km, respectively.

We conduct the experiments on two public datasets, the Oxford Radar RobotCar (RobotCar) dataset (Maddern et al., 2017; Barnes et al., 2020a) and the Multimodal Range (MulRan) dataset (Kim et al., 2020). Both these datasets use the Navtech FMCW radar but the 3D lidar sensors use different ones, double Velodyne HDL-32E and one Ouster OS1-64. Our proposed lidar submap construction is able to reduce the differences of the two lidar equipments and settings on mobile robots. To demonstrate the generalization ability, we follow the training strategy of our previous work (Yin et al., 2021), in which only part of the RobotCar dataset is used for training. In the test stage, as shown in Figure 3, the learned model is evaluated on another part of the RobotCar and also generalized to MulRan-Riverside and MulRan-KAIST directly without retraining.

Despite the cross-dataset above, the multi-session evaluation is also used for validation. Specifically, for RobotCar and MulRan datasets, we use one sequence or session as a map database and then select another session on a day as query data. The selected sessions are presented in Table 1.

**Table 1:** Sequences for training and testing.

**Dataset**	**Date**	**Length (km)**	**Usage**
Oxford Radar RobotCar	10/01/2019	9.02	Training and map database
Oxford Radar RobotCar	11/01/2019	9.03	Query data for test
MulRan-Riverside	16/08/2019	6.61	Map database
MulRan-Riverside	02/08/2019	7.25	Query data for test
MulRan-KAIST	23/08/2019	5.97	Map database
MulRan-KAIST	20/06/2019	6.13	Query data for test

To better explore parameter sensitivity, we conduct experiments using various distance thresholds and margin values in $L_{1}$. Firstly, we set *m* = 1.0 as a constant value and train the proposed learning model. Then, we set different distance thresholds *d* for evaluation on MulRan-KAIST, which means a found pair of places is considered as true positive when its distance is below *d* meters. Specifically, we use recall@1 to evaluate the performance, which is calculated as follows:

(4) $$\begin{array}{l} \text{recall@1}=\frac{\text{true positive samples}}{\text{number of query scans}}\% \end{array}$$

**Figure 4:**
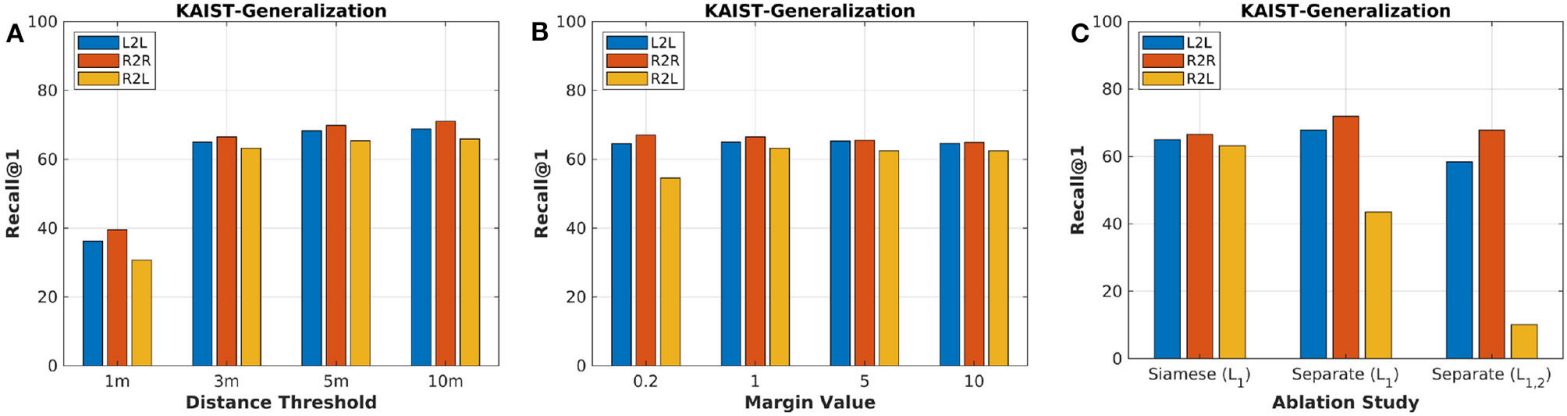
**(A)** The parameter sensitivity study of distance thresholds for evaluation. **(B)** The parameter sensitivity study of margin values for training. **(C)** The ablation study of proposed framework and loss functions.

As a result, the sensitivity of distance thresholds is shown in Figure 4A. The higher threshold, the better performance of the trained model. We select 3 m as the distance threshold for all the following tests, which was also conducted in our previous work (Yin et al., 2019). Furthermore, we change the margin values and train models. The test result is shown in Figure 4, and our model achieves the best R2L performance when *m* = 1.

In this paper, we propose to extract feature embeddings *via* Siamese neural network. One might suggest that the two embeddings of lidar and radar should be achieved with two individual networks. To validate the proposed framework, we conduct ablation study for the framework structure. Firstly, we abandon the Siamese network N and train two separate encoder-decoders *via* joint learning and loss function $L_{1}$. Secondly, we propose to train this new framework with another transformation loss function $L_{2}$ together, formulated as follows:

(5) $$\begin{array}{l} L_{2}=\frac{1}{\left|F\right|}\underset{F\in F}{\sum }∥F_{R}-F_{L}∥ \end{array}$$

(6) $$\begin{array}{l} L_{1,2}=L_{1}+\alpha L_{2} \end{array}$$

where *F*_*R*_ and *F*_*L*_ are generated signatures of radar and lidar, and we set α = 0.2 to balance the triplet loss and transformation loss. Figure 4C presents the experimental results with different structures and loss functions. Although the framework with two individual networks performs better than the Siamese one on R2R and L2L, the shared network matches more correct R2L for the heterogeneous place recognition. The transformation loss seems to be redundant for the learning task in this paper. Based on the ablation analysis above, we use the proposed method in section 3 for the following evaluation and comparison.

### 4.2. Single-Session: Loop Closure Detection

Single-session contains one driving or traveling data collected by the mobile platform. We evaluate the online place recognition performance on a single session, which equals to the loop closure detection for a mapping system. Generally, one single sensor is sufficient to build maps considering the consistency of the sensor modality. In this context, R2L evaluation is unnecessary, and we perform R2R on MulRan-KAIST to validate the homogenous place recognition.

**Figure 5:**
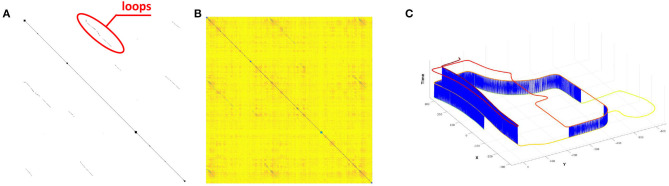
**(A)** The ground truth based binary matrix, where the black points are the loops. **(B)** The R2R based similarity matrix generated using the proposed method. **(C)** The loops closure detection result under certain threshold, and the blue lines are the detected loops. Note that a growing height and a color-changing are added to each pose with respect to the time for visualization.

We compute similarity matrix (Yin et al., 2017) and obtain the loop closures under certain threshold. The black points are marked as loop closures in Figure 5A, and the darker pixels are with higher probabilities to be loop closures in Figure 5B. It is clear that there are true positive loops in the similarity matrix, and a number of loops can be found *via* thresholding. In Figure 5C, the visualization result shows that our proposed method is able to detect radar loop closures when the vehicle is driving in opposite directions. Overall, the qualitative result of online place recognition demonstrates that our proposed method is feasible for consistent map building. While for the global localization, multi-session place recognition is required, and we conduct quantitative experiments as follows.

### 4.3. Multi-Session: Global Localization

In this sub-section, we evaluate the place recognition performance on multi-session data. Specifically, the first session is used as the map database, and the second session is regarded as the query input, thus achieving global localization across days.

The proposed joint learning-based method is compared to other two competitive methods. First, the ScanContext is used for comparison, which achieves not only 3D lidar place recognition (Kim and Kim, 2018) but also 2D radar place recognition in the recent publication (Kim et al., 2020). Secondly, the DiSCO method (Xu et al., 2021) is implemented as another comparison, and the quadruplet loss term is used in the learning stage. DiSCO is not designed for heterogeneous place recognition, so we train two models for L2L and R2R separately and test the R2L using the signatures from these models. On the other hand, DiSCO can be regarded as the model without the joint learning in this paper. Finally, for making a fair comparison, we use the lidar submaps as input for all the methods in this paper.

**Figure 6:**
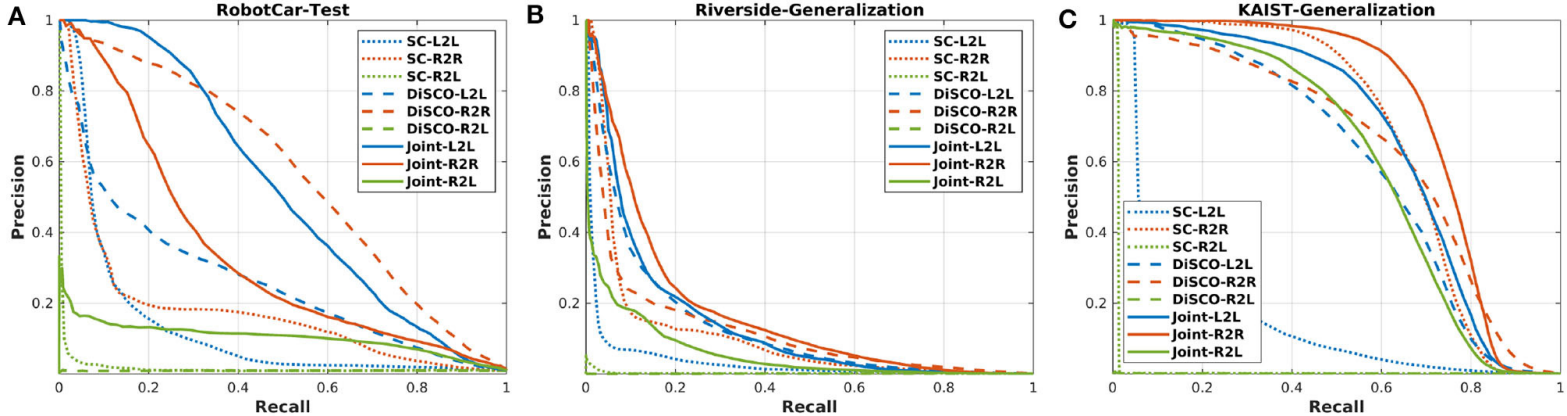
**(A)** The precision-recall curves of the RobotCar testing session. **(B,C)** The model trained from RobotCar dataset is generalized to MulRan-Riverside and MulRan-KAIST.

Figure 6 presents the precision-recall curves, which are generated from the similarity matrices compared to the ground truth-based binary matrices. Since the computing of similarity matrix is much more time consuming for ScanContext, we only present the precision-recall curves for DiSCO and our proposed method. In addition, we also provide maximum *F*_1_ scores in Table 2, and recall@1 results in Table 3, which is based on how many correct top-1 can be found using place recognition methods. In Kim and Kim (2018) and Kim et al. (2020), the top-1 is searched with a coarse-to-fine strategy, and we set the number of coarse candidates as 1% of the database.

**Table 2:** Maximum *F*_1_ score of precision-recall curves.

**Dataset**	**RobotCar-Test**			**MulRan-Riverside**			**MulRan-KAIST**		
	**L2L**	**R2R**	**R2L**	**L2L**	**R2R**	**R2L**	**L2L**	**R2R**	**R2L**
ScanContext, Kim et al. (2020)	0.18	0.24	0.04	0.08	0.16	0.02	0.24	0.67	0.02
DiSCO, Xu et al. (2021)	0.33	**0.56**	0.02	0.20	0.20	0.00	0.59	0.63	0.01
Joint Learning	**0.50**	0.35	**0.18**	**0.21**	**0.22**	**0.14**	**0.66**	**0.74**	**0.61**

*The bold values are the biggest number in each column respectively, which means that one method achieves better performance compared to the other two methods for L2L, R2R or R2L*.

**Table 3:** Recall@1 (%) of multi-session place recognition.

**Dataset**	**RobotCar-Test**			**MulRan-Riverside**			**MulRan-KAIST**		
	**L2L**	**R2R**	**R2L**	**L2L**	**R2R**	**R2L**	**L2L**	**R2R**	**R2L**
ScanContext, Kim et al. (2020)	92.51	91.35	1.33	37.22	42.68	1.17	28.81	**72.65**	0.55
DiSCO, Xu et al. (2021)	89.68	93.01	1.66	**42.19**	**44.08**	0.99	**66.77**	70.86	0.33
Joint Learning	**93.68**	**93.18**	**61.23**	38.89	41.15	**26.20**	65.01	66.56	**63.22**

*The bold values are the biggest number in each column respectively, which means that one method achieves better performance compared to the other two methods for L2L, R2R or R2L*.

As a result, in Figure 6 and Table 3, our proposed method achieves comparable results on R2R and L2L, and also on R2L application, which is the evaluation result based on lidar database and radar query. As for ScanContext and DiSCO, both two methods achieve high performance on L2L and R2R, but radar and lidar are not connected to each other in these methods, resulting in a much lower performance on R2L. We also note that ScanContext performs much worse with MulRan-KAIST, in which many dynamical objects exist. The other two learning-based methods are able to handle these challenging environments. Overall, the multi-session place recognition results demonstrate that our proposed method achieves both homogeneous and heterogeneous place recognition, and our model requires less training stage compared to DiSCO.

### 4.4. Case Study

**Figure 7:**
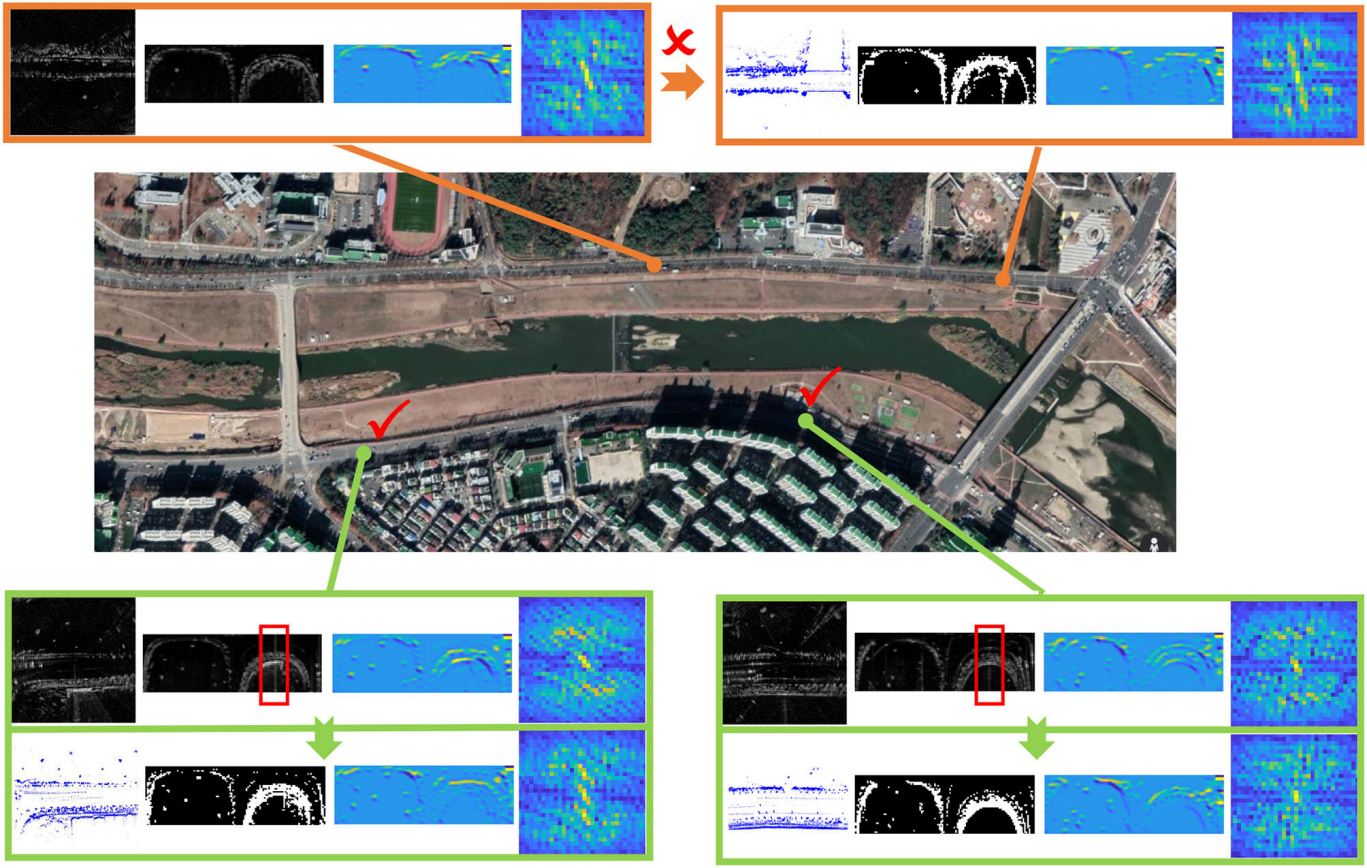
Case study examples on radar-to-lidar (R2L) place recognition, where the lidar database and radar query are collected in different days. We also present ScanContext representations, feature embeddings, and final signatures. Some false positives by saturation are also marked in red boxes.

Finally, we present several case study examples on the challenging MulRan-Riverside, where many structural features are repetitive along the road. As shown in Figure 7, the trained model results in a failed case since the bushes and buildings are quite similar to two different places, and in this context, the radar from the query data is matched to the wrong lidar-based database. As for the two correct cases, there exist specific features on the sides of streets, corners, and buildings, etc., thus making the place recognition model more robust in these challenging environments.

In Figure 7, it is obvious that the semantics near the roads are kept in the feature embeddings, which are enhanced *via* the joint learning in this paper. We also note that there are false positive noises by saturation (in red boxes), but the noises are removed in the learned feature embeddings, thus demonstrating the effectiveness of the proposed joint learning paradigm.

## 5. Conclusion

In this paper, we propose to train a joint learning system for radar and lidar place recognition, which helps the robot recognize the revisited lidar submaps using current radar scan. Specifically, we first encode the radar and lidar points based on the ScanContext, then we build a shared U-Net to transform the handcrafted features to the learned representations. To achieve the place recognition, we also apply the triplet loss as the supervision. The whole system is trained jointly with both lidar and radar input. Finally, we conduct the training and testing on the public Oxford RobotCar dataset and also the generalization on MulRan dataset. Compared to the existing place recognition methods, our proposed framework achieves not only single-sensor based place recognition but also the heterogeneous place recognition of (R2L), demonstrating the effectiveness of our proposed joint learning framework.

Despite the conclusions above, we also consider there still remain several promising directions for heterogeneous measurement-based robotic perception. Firstly, the submap building is critical for the heterogeneous place recognition in this paper, which can be improved with a more informative method (Adolfsson et al., 2019). Secondly, we consider a Global Positioning System (GPS)-aided or sequential-based place recognition is desired for real applications, thus making the perception system more efficient and effective in the time domain. Finally, we consider the integration of place recognition and pose estimator, Monte Carlo localization, for example (Yin et al., 2019; Sun et al., 2020), which is a good choice for metric robot localization.

## Data Availability Statement

The original contributions presented in the study are included in the article/supplementary material, further inquiries can be directed to the corresponding author/s.

## Author Contributions

HY: methodology, implementation, experiment design, visualization, and writing. XX: methodology and review. YW: analysis, supervision, and review. RX: supervision and project administration. All authors contributed to the article and approved the submitted version.

## Conflict of Interest

The authors declare that the research was conducted in the absence of any commercial or financial relationships that could be construed as a potential conflict of interest.

## Footnotes

^1^https://navtechradar.com
